# Association between Toll-Like Receptor 4 Polymorphisms and Systemic Lupus Erythematosus Susceptibility: A Meta-Analysis

**DOI:** 10.1155/2016/7842587

**Published:** 2016-08-29

**Authors:** Weiping Hu, Senchao Wu, Yanlin Zhang, Keshav Raj Sigdel, Yong Lin, Hongbin Zhong

**Affiliations:** ^1^Department of Nephrology, First Affiliated Hospital of Xiamen University, Xiamen, Fujian 361003, China; ^2^Department of Nephrology, Longyan First Hospital Affiliated to Fujian Medical University, Longyan, Fujian 36400, China; ^3^Arogya Health Home, Arthritis and Rheumatic Diseases Treatment Center, Jawalakhel, Lalitpur, Nepal; ^4^Department of Infectious Disease Control, Xiamen Center for Disease Control and Prevention, Xiamen, Fujian 361021, China; ^5^Department of Nephrology, Xiamen City Fifth Hospital, Xiamen, Fujian 361101, China

## Abstract

Family aggregation was observed among systemic lupus erythematosus (SLE) cases, suggesting the genetic factor may contribute to the susceptibility. Toll-like receptors (TLR) play key role in human immune system; in order to gain better insight on the association between TLR4 polymorphisms and SLE risk, a meta-analysis was conducted. In total 4 case-control studies have been included, involving 503 SLE cases and 636 healthy controls. The association between TLR4 polymorphisms and SLE risk was evaluated by calculating pooled odd ratio (OR) and its 95% confidential interval (CI). The *Q*-test and *I* ^2^ statistic were used to estimate the degree of heterogeneity. Publication bias among enrolled studies was examined by using Egger's test and Begg's test. Overall, there was no evidence of positive association between SLE risk and D299G and T399I polymorphisms in TLR4. The meta-analysis reported a null association between TLR4 polymorphisms and SLE risk in included study populations, but the role of TLR4 polymorphisms in developing SLE among other populations remains undetermined. Moreover, some laboratory studies still discovered the involvement of TLR4 in SLE process. Therefore, the association between TLR4 polymorphisms and SLE risk requires further investigation both in laboratory and in epidemiological efforts.

## 1. Introduction

Systemic lupus erythematosus is an autoimmune disease that causes a chronic inflammatory condition for life-long time, and the inflammation triggered by SLE can affect multiple organs in human body, including joints, skin, lung, kidneys, and blood vessels. Particularly the impairment of kidneys may lead to lupus nephritis and possibly develop to acute or end-stage renal failure. Although some of SLE complications could be fatal, the development and application of immunosuppressive drugs aiming to control the autoimmune process helped to improve the prognosis of SLE cases; approximately 80–90% of SLE cases can expect to have a normal life expectancy [1]. However, due to the wide spectrum of clinical manifestation SLE caused, it may be very difficult for diagnosis unless with professional opinion of rheumatologist. Therefore, the early diagnosis of SLE would be critical to improve the prognosis and life quality of SLE cases. Besides, SLE has a unique pattern of incidence, it is more common in female population than male counterparts, and the gender ratio ranges from 4 to 12 : 1. The SLE could occur in all ages, but the incidence peak appears in childbearing years, according to statistics [2]. With years of endeavor in surveillance programs and SLE registry, the prevalence of SLE in some states of United States has been well documented. According to the data released by the Michigan Lupus Epidemiology and Surveillance Program, the incidence by American College of Rheumatology (ACR) definition among black female was the highest when comparing with other races, reaching 12.8 per 100,000 (95% CI: 11.1–14.8), and the incidence of white female was approximately half of the corresponding indicator of black female; to be more accurate, the figure was 6.3 (95% CI: 5.3–7.5) [3]. Consistent with the previous findings, the data reflects that race would affect the incidence and prevalence of SLE, and the prevalence of SLE is higher among African descendants, Asians, and Hispanic descendants, when comparing with white population. The family history of SLE has also been recognized as the risk factor of developing SLE; an epidemiological study conducted among 265 SLE cases and 355 healthy controls demonstrated that family history of SLE would confer an elevated risk with an OR of 3.3 (95% CI: 1.2–8.6) [4].

Although many studies have been done to investigate the risk factors of SLE, the cause of SLE has not yet been fully understood so far. Based on the family aggregation observed, it can be assumed that genetic factor plays a critical role in developing SLE. No single causal gene has been identified so far, but several genes have been proved to influence individual's chance of developing SLE. A study conducted in Caucasian population demonstrated that the human leukocyte antigen classes I, II, and III are associated with SLE risk [5]. Similar with HLA regions, toll-like receptors are a class of proteins that play a fundamental role in the early innate immune response to pathogens by sensing microorganism and are involved in detecting endogenous danger signals. The animal experiment showed that, among the mice with TLR2 and TLR4 deficiency, the immunological alteration and autoantibody production have been significantly suppressed, suggesting the TLR4 is involved with the autoimmune process [6]. Therefore, the polymorphisms in TLR4 could possibly affect the SLE risk among population; however, there is no conclusion reached in the association between TLR4 polymorphisms and SLE risk. In order to analyze the impact of TLR4 polymorphisms and gain comprehensive insight on this issue, we conducted a meta-analysis.

## 2. Material and Methods

We performed the meta-analysis in accordance with the Preferred Reporting Items for Systematic Reviews and Meta-Analyses (PRISMA) guidelines [7].

### 2.1. Identification and Eligibility of Relevant Studies

Literature search was independently conducted by two investigators for genetic studies on TLR4 in PUBMED. All relevant studies reported up to January 2015 and following key words were searched: “systemic lupus erythematosus,” or “SLE”, “polymorphism” or “SNP”, “toll-like receptor 4,” or “TLR4”. The search was performed in February 2016 and no date and language limits were applied. In order to further expand the dataset, we also reviewed the reference lists of all retrieved studies to identify eligible studies.

### 2.2. Inclusion and Exclusion Criteria

The eligibility of study was defined as follows: (1) case-control study design; (2) investigating the association between TLR4 polymorphisms and the risk of developing SLE; (3) genotype distribution of TLR4 polymorphisms in both cases and controls available; reviews, cases reports, editorial comment, communications, and reports without sufficient data were excluded in our meta-analysis.

### 2.3. Data Extraction and Quality Assessment

The following information was extracted from each eligible study: name of first author, year of publication, ethnicity of study participants, number of cases and controls, the frequency of TLR4 genotypes in SLE cases and controls, and Hardy-Weinberg equilibrium (HWE) test results in controls. In order to assess the quality of enrolled studies, we rated the methodological quality of each included study by using the Newcastle-Ottawa quality assessment scale. This scale contains 9 items in total (1 point for each) in three parts: selection (4 items), comparability (2 items), and exposure (3 items).

### 2.4. Statistical Analysis

Statistical analysis was performed by using STATA version 12 (StataCorp, College Station, TX, USA). Chi-squared test was employed to determine the HWE of controls if the *P* value of HWE was not provided in the original study. A *P* value less than 0.05 was considered as the deviation of HWE. The association between TLR4 polymorphisms and SLE risk was evaluated by calculating pooled odd ratio and its 95% confidential interval and forest plot was created to demonstrate the overall effect. With respect to heterogeneity, the *Q*-test and *I* ^2^ statistic were used to estimate the degree of heterogeneity among the enrolled studies. In the absence of heterogeneity, fixed-effects model was applied to estimate the pooled OR and 95% CI. Otherwise, random-effect model was used to yield more conservative overall effects. Moreover, publication bias among enrolled studies was examined by using Egger's test and Begg's test, where a *P* value of less than 0.10 was considered statistically significant.

## 3. Results

### 3.1. Characteristics of Enrolled Studies

**Figure 1:**
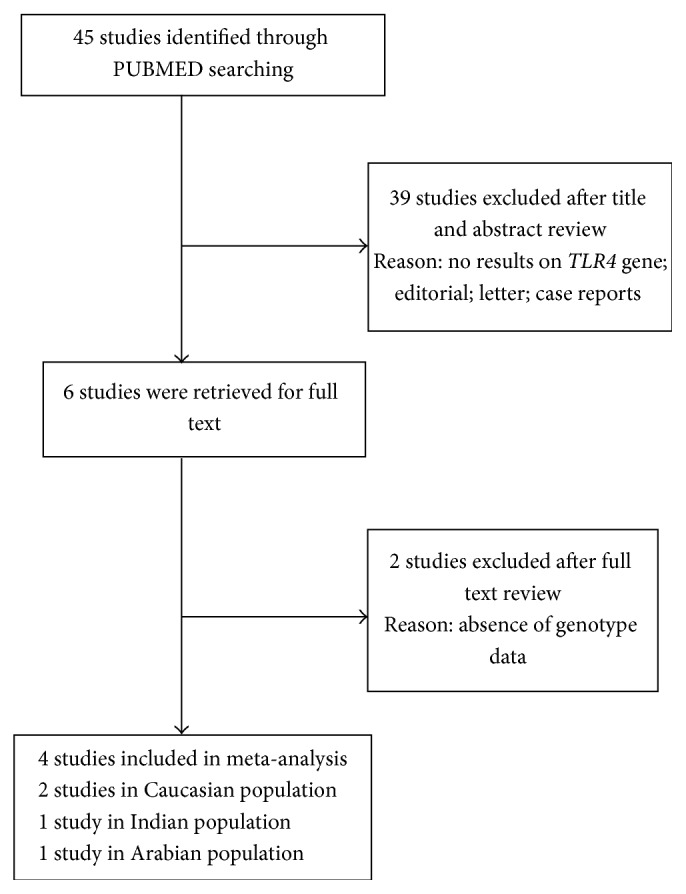
PRISMA flow diagram for inclusion of the studies investigating the relationship between* TLR4* polymorphisms and SLE risk.

As shown in Figure 1, a detailed flow diagram demonstrated the process of inclusion and exclusion in this meta-analysis. Based on our search strategy, a total of 45 studies were identified in the initial search. After studying the abstracts, 39 publications have been removed. After reviewing the full text of the remaining 6 studies, 2 publications have been excluded due to the absence of genotype distribution data. Finally, a total of 4 studies were included in our meta-analysis [8–11], involving 503 SLE cases and 636 healthy controls. The characteristics of enrolled studies were demonstrated in Table 1. Of 4 studies enrolled, 2 were conducted in Caucasian population, 1 in Arabian, and 1 in Indian. The genotype distribution of both D299G and T399I polymorphisms agreed on HWE in control group in every study. The average of quality score evaluated by Newcastle-Ottawa scale was 7.2 combined with all enrolled studies together, while a score greater than 5 was considered appropriate to be included in meta-analysis.

### 3.2. Meta-Analysis of TLR4 Polymorphisms and SLE Risk

**Figure 2:**
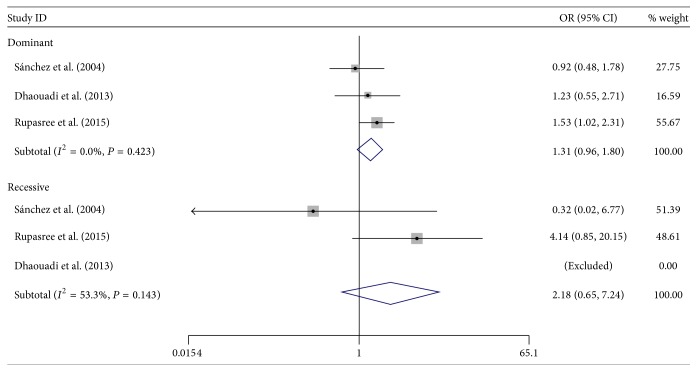
Forest plot of the association between* TLR4* polymorphism D299G and SLE risk.

**Figure 3 fig3:**
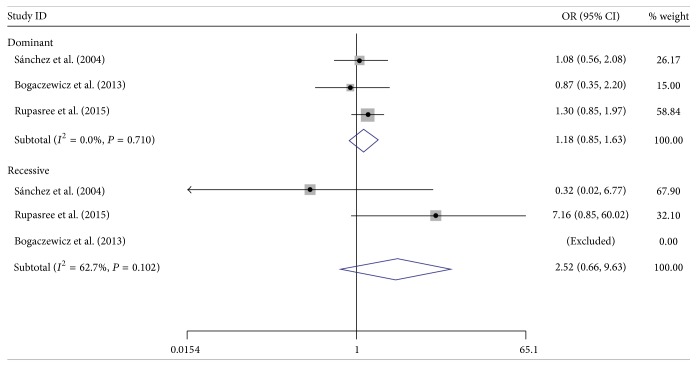
Forest plot of the association between* TLR4* polymorphism T399I and SLE risk.

**Figure 4:**
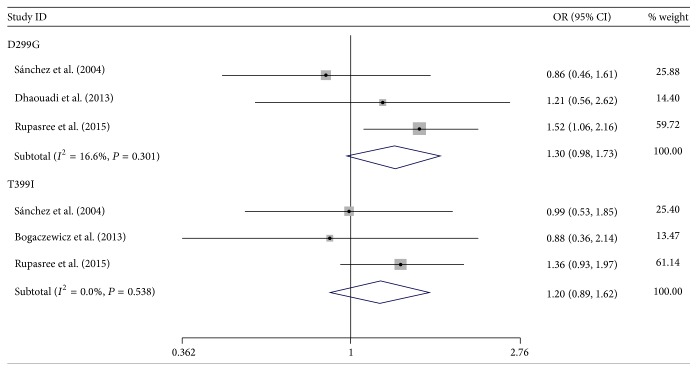
Forest plot of the association between D299G, T399I allele, and SLE risk.

The main outcomes of this meta-analysis were presented in Table 3 and the genotype distribution in cases and controls from enrolled studies was presented in Table 2. Overall, there was no evidence of positive association between SLE risk and TLR4 D299G and T399I polymorphisms. As for D299G, the pooled ORs in dominant model and recessive model were 1.31 (95% CI: 0.96–1.80) and 2.18 (95% CI: 0.65–7.24), respectively. The pooled OR for T399I was 1.18 (95% CI: 0.85–1.64) in dominant model, and the corresponding figure was 2.52 (95% CI: 0.66–9.63) in recessive model. We further analyzed the association between alleles of these two TLR4 polymorphisms and SLE risk by pooling all subjects; the pooled ORs for D299G and T399I allele were 1.30 (95% CI: 0.98–1.73) and 1.20 (95% CI: 0.89–1.62), respectively (see Figures 2 [Fig fig3]–4).

### 3.3. Evaluation of Heterogeneity

According to the *I* ^2^ statistics we calculated by using STATA software, there was no interstudy heterogeneity among all enrolled studies of TLR4 polymorphisms for all 3 genetic models (D299G dominant model: *I* ^2^ = 0.0%, *P* = 0.432; recessive model: *I* ^2^ = 53.3%, *P* = 0.143; allele: *I* ^2^ = 16.6%, *P* = 0.301; T399I dominant model: *I* ^2^ = 0.0%, *P* = 0.710; recessive model: *I* ^2^ = 62.7%, *P* = 0.102; allele: *I* ^2^ = 0.0%, *P* = 0.538). Due to the absence of heterogeneity, the pooled ORs were estimated by using fixed-effect model.

### 3.4. Publication Bias

Due to the limited number of enrolled studies, it was inappropriate to evaluate the publication bias by using funnel plot. Therefore, the publication bias in our meta-analysis was estimated by using Begg and Egger's test. As shown in Table 4, the *P* values for all genetic models were all greater than 0.05, suggesting no evidence of publication bias.

## 4. Discussion

Toll-like receptors were firstly discovered in* Drosophila* in 1985 and they have been proved to play fundamental role in innate immune system. After that, Nomura et al. identified the first toll-like receptor in cDNA clones of human in 1994 [12]. By comparing the sequence, it has been discovered that TLRs are highly conservative from* Drosophila* to human and share structural and functional similarities. The main function of TLR4 is recognizing lipopolysaccharide (LPS) and activating immune response; previous findings also revealed that TLR4 is capable of binding a variety of endogenous proteins such as low-density lipoprotein and heat shock protein [13]. With the initiation of LPS, TLR4 is capable of inducing the expression of multiple cytokines, including IL-1, IL-6, and IL8 via signaling pathway. The relationship between TLR4 and infection has been widely investigated; after initial TLR mediated immune response triggered by LPS, it is possible for secondary response such as activation of endothelial cells that promotes the production of adhesion molecules to occur [14]. Eventually it may lead to systemic septic syndrome including tissue perfusions and organ failure [15]. Given the great importance of TLR4 in immune response, growing evidence implicates the association between TLR4 and autoimmune condition [16].

The mutation occurring in TLR4 sequence may also alter individual's susceptibility to some diseases. In detail, two nonsynonymous polymorphisms in TLR4 gene have been well investigated; an A/G transition causes an Asp/Gly polymorphism at amino acid 299 and a C/T transition causes a Thr/Ile polymorphism at amino acid 399. Notably, the amino acid change caused by two above-mentioned polymorphisms would lead to changing the ligand-binding site of the receptor [17] and consequently contributes to the alternation of susceptibility to diseases. One of the underlying mechanisms is that the above-mentioned two polymorphisms may have impact on the responsiveness to LPS. To be more specific, the extracellular domain of TLR4 can bind with a secreted protein named MD-2 [18] and a soluble or GPI-anchored glycoprotein coreceptor CD14 which are essential for optimal TLR-4 mediated LPS response [19]. According to the results of sequencing, both D299G and T399I are located in the extracellular domain of TLR4, and these two variants have been associated with LPS hyporesponsiveness* in vitro* and* in vivo* [20]. A much debated question is how D299G and T399I affect the LPS responsiveness and the result remains inconsistent. A study which enrolled 200 plus pediatric subjects and quantification of pro- and anti-inflammatory cytokines response by fresh peripheral blood mononuclear cell upon acute exposure to LPS were conducted, and the results showed no significant difference between subjects with different genotype of TLR4 polymorphisms, indicating a null association [21]. Although the previous study reported that the polymorphisms in TLR4 have no impact on the production of cytokines, a more recent cell assay suggested that the polymorphisms in TLR4 did not alter LPS-binding to soluble TLR4/MD2; instead, the native-PAGE revealed that D299G and T399I could impair the ligand-dependent dimerization and consequently lead to the hyporesponsiveness to LPS [22]. In a case-control study involving 108 Legionnaire's disease cases and two independent control groups, both polymorphisms were inversely associated with the risk of Legionnaire's disease [23]. However, the issue of TLR4 polymorphisms and disease risk still remains controversial; another study revealed that the mutation of TLR4 is positively associated with the risk of developing leprosy [24]. So far there is only a limited number of studies that investigated the association between TLR4 polymorphisms and SLE risk; null association was reported in the case-control study in Spanish and Polish population [8, 9], while a significant association was observed in Indian population [11]. Therefore, we conducted a comprehensive literature search and meta-analysis to address this issue. According to the *I* ^2^ statistics among all genetic models, it can be assumed that the heterogeneity was insignificant in our meta-analysis; therefore, fix-effect model was employed to estimate the pooled OR. Both D299G and T399I were not significantly associated with SLE risk in dominant and recessive model. We further analyzed the overall impact of TLR4 polymorphisms allele on SLE risk, and the results were similar with the genotype analysis. Therefore, we can conclude that, based on the current evidence we obtained, the TLR4 polymorphisms are not associated with SLE risk.

The major limitation of our meta-analysis is that the number of enrolled studies was small; despite expanded search applied by reviewing the reference of enroll studies, still only 4 studies were available in our final analysis. The reason behind this problem is the unique distribution pattern of TLR4 polymorphisms between different races. According to the data of a study involving study subjects from all over the world, the D299G and T399I double mutation haplotype were of high frequency in Caucasians in Europe, and D299G and 399 wildtype can be found in African population; however, no mutations in both D299G and T399I were observed in East Asian countries, including China, Japan, and Korea [25]. Therefore, the range of study population was limited by the natural genotype distribution. Despite the limitation of the above-mentioned, the pooled outcome of this meta-analysis is solid, due to the absence of both heterogeneity and publication bias.

Although the meta-analysis we conducted reported a null association between TLR4 polymorphisms and SLE risk, some laboratory studies still discovered the involvement of TLR4 in SLE process. Flow cytometry was applied to detect the expression of TLR4 in blood monocytes from SLE cases and healthy control; the statistical comparison revealed that the TLR4 expression was significantly suppressed in SLE cases [26]. Moreover, the present meta-analysis only included Caucasian, Arabian, and Indian due to the data availability; therefore, the conclusion does not apply to all populations. Recently, TLR4 has been recognized as the potential target of novel therapy of SLE, and progress has been made. Therefore, the association between TLR4 polymorphisms and SLE risk requires further investigation both in laboratory and in epidemiological efforts.

## Figures and Tables

**Table 1:** Characteristics of enrolled studies in the meta-analysis on the association between TLR4 polymorphisms and SLE risk.

Author and year	Ethnicity	Quality score	*P*_HWE_ in control (D299G)	*P*_HWE_ in control (T399I)
Sánchez, 2004 [8]	Caucasian	8	0.78	0.27
Bogaczewicz, 2013 [9]	Caucasian	6	N/A	0.42
Dhaouadi, 2013 [10]	Arabian	8	0.55	N/A
Rupasree, 2015 [11]	Indian	7	0.28	0.22

**Table:** (a) D299G

Author and year	Case				Control			
	Asp/Asp	Asp/Gly	Gly/Gly	Total	Asp/Asp	Asp/Gly	Gly/Gly	Total
Sánchez, 2004 [8]	106	16	0	122	171	26	2	199
Bogaczewicz, 2013 [9]	—	—	—	—	—	—	—	—
Dhaouadi, 2013 [10]	111	16	0	127	102	12	0	114
Rupasree, 2015 [11]	119	68	7	194	158	63	2	223

**Table:** (b) T399I

Author and year	Case				Control			
	Thr/Thr	Thr/Ile	Ile/Ile	Total	Thr/Thr	Thr/Ile	Ile/Ile	Total
Sánchez, 2004 [8]	105	17	0	122	173	24	2	199
Bogaczewicz, 2013 [9]	52	8	0	60	85	15	0	100
Dhaouadi, 2013 [10]	—	—	—	—	—	—	—	—
Rupasree, 2015 [11]	128	58	6	192	161	61	1	223

**Table 3:** Meta-analysis of *TLR4* polymorphism with the risk of SLE.

Polymorphism	Genetic model	Test of association			Test of heterogeneity		
		OR	95% CI	*P*	Model	*I* ^2^	*P*
D299G	Dominant	1.31	0.96, 1.80	0.09	Fixed	0.0%	0.432
	Recessive	2.18	0.65, 7.24	0.21	Fixed	53.3%	0.143
	Allele	1.30	0.98, 1.73	0.07	Fixed	16.6%	0.301

T399I	Dominant	1.18	0.85, 1.64	0.33	Fixed	0.0%	0.710
	Recessive	2.52	0.66, 9.63	0.18	Fixed	62.7%	0.102
	Allele	1.20	0.89, 1.62	0.24	Fixed	0.0%	0.538

**Table 4:** The results of publication bias in dominant and recessive model and allele analysis.

Polymorphism	Genetic model	Begg's test		Egger's test	
		*Z*	*P*	Coefficient	*P*
D299G	Dominant	−0.52	0.60	−2.03	0.45
	Recessive	−1.00	0.32	−3.42	0.24
	Allele	−0.52	0.60	−2.09	0.45

T399I	Dominant	−1.57	0.12	−1.15	0.56
	Recessive	−1.00	0.32	−2.15	0.32
	Allele	−1.57	0.12	−1.83	0.15
